# Rare Superior Mesenteric Artery Syndrome in an Eight-Year-Old Girl With Henoch-Schönlein Purpura: A Case Report

**DOI:** 10.7759/cureus.69389

**Published:** 2024-09-14

**Authors:** Yamen Shayah, Osama Almadhoun, Drew Pierce, John H Lillvis, Rabheh Abdul-Aziz

**Affiliations:** 1 Medical Education, Alfaisal University College of Medicine, Riyadh, SAU; 2 Pediatric Gastroenterology, University at Buffalo Jacobs School of Medicine and Biomedical Sciences, Buffalo, USA; 3 Pediatric Radiology, University at Buffalo Jacobs School of Medicine and Biomedical Sciences, Buffalo, USA; 4 Pediatric Ophthalmology, University at Buffalo Jacobs School of Medicine and Biomedical Sciences, Buffalo, USA; 5 Pediatric Rheumatology, University at Buffalo Jacobs School of Medicine and Biomedical Sciences, Buffalo, USA

**Keywords:** s: superior mesenteric artery syndrome, steroid use, gastrointestinal complication, pediatric onset vasculitis, henoch schönlein purpura

## Abstract

Henoch-Schönlein purpura (HSP) is a systemic vasculitis characterized by palpable purpura, arthralgia or arthritis, GI symptoms, and renal involvement. Superior mesenteric artery (SMA) syndrome, a rare condition, occurs when the third part of the duodenum is compressed between the aorta and the SMA, leading to upper intestinal obstruction. This case report describes the clinical presentation, diagnostic process, and management of an eight-year-old girl with HSP complicated by SMA syndrome. The patient initially presented with abdominal pain and vomiting, eventually developing the characteristic rash of HSP. While initial management was supportive, her condition deteriorated. Treatment with intravenous methylprednisolone resulted in significant symptom improvement and resolution of both SMA syndrome and HSP manifestations. This case highlights the need to recognize SMA syndrome as a potential complication of HSP and demonstrates the effectiveness of steroid therapy in managing this condition. Further research is needed to develop comprehensive treatment guidelines for HSP patients with SMA syndrome.

## Introduction

Henoch-Schönlein purpura (HSP) is a common childhood systemic vasculitis marked by cutaneous palpable purpura, arthralgia or arthritis, GI involvement, and kidney involvement [1]. Also known as immunoglobulin A (IgA) vasculitis, HSP is characterized by the deposition of IgA1-immune complexes, complement factors, and neutrophil infiltration, which contribute to vascular inflammation [2]. The incidence of HSP in children ranges from 10 to 20 cases per 100,000 annually, with 90% of cases occurring between ages two and 10 [3].

Superior mesenteric artery (SMA) syndrome occurs when the third part of the duodenum is compressed between the aorta and the SMA, leading to upper intestinal blockage. Diagnosis is typically made through radiologic studies. This rare condition is often associated with underlying medical conditions linked to catabolic states, rapid weight loss, or surgeries that alter anatomy. Conservative treatment is usually effective [4].

The intersection of HSP and SMA syndrome is not well documented. A review of the literature reveals only one case report and one case-control study by Harada et al. (2007, 2011), which described duodenal abnormalities in HSP that mimicked SMA syndrome [5,6]. This suggests a potential, although unrecognized, link between the two conditions.

In this case report, we present an eight-year-old patient with HSP who developed SMA syndrome as a result of duodenal inflammation. Our findings align with those of Harada et al., highlighting that SMA syndrome can indeed present as part of the clinical spectrum of HSP [5]. By sharing this case, we aim to raise awareness of this rare but significant association and provide insights into the management of such cases.

## Case presentation

**Figure 1:**
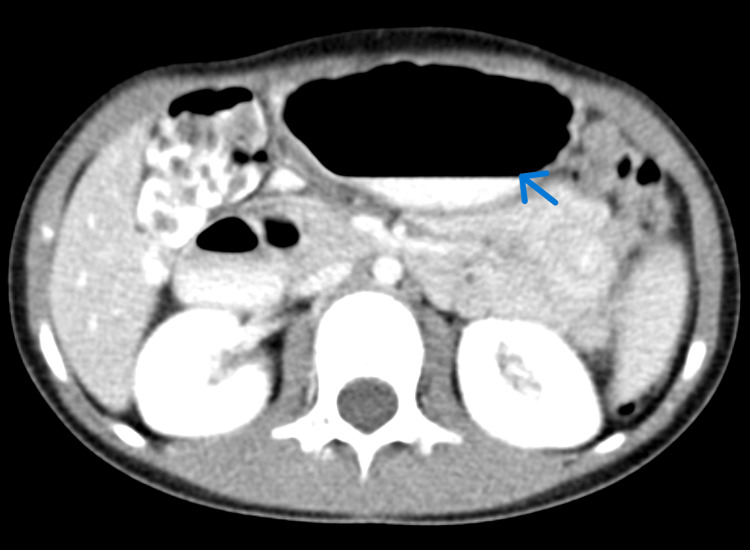
Axial CT showing dilated proximal duodenum with an air-fluid level

**Figure 2:**
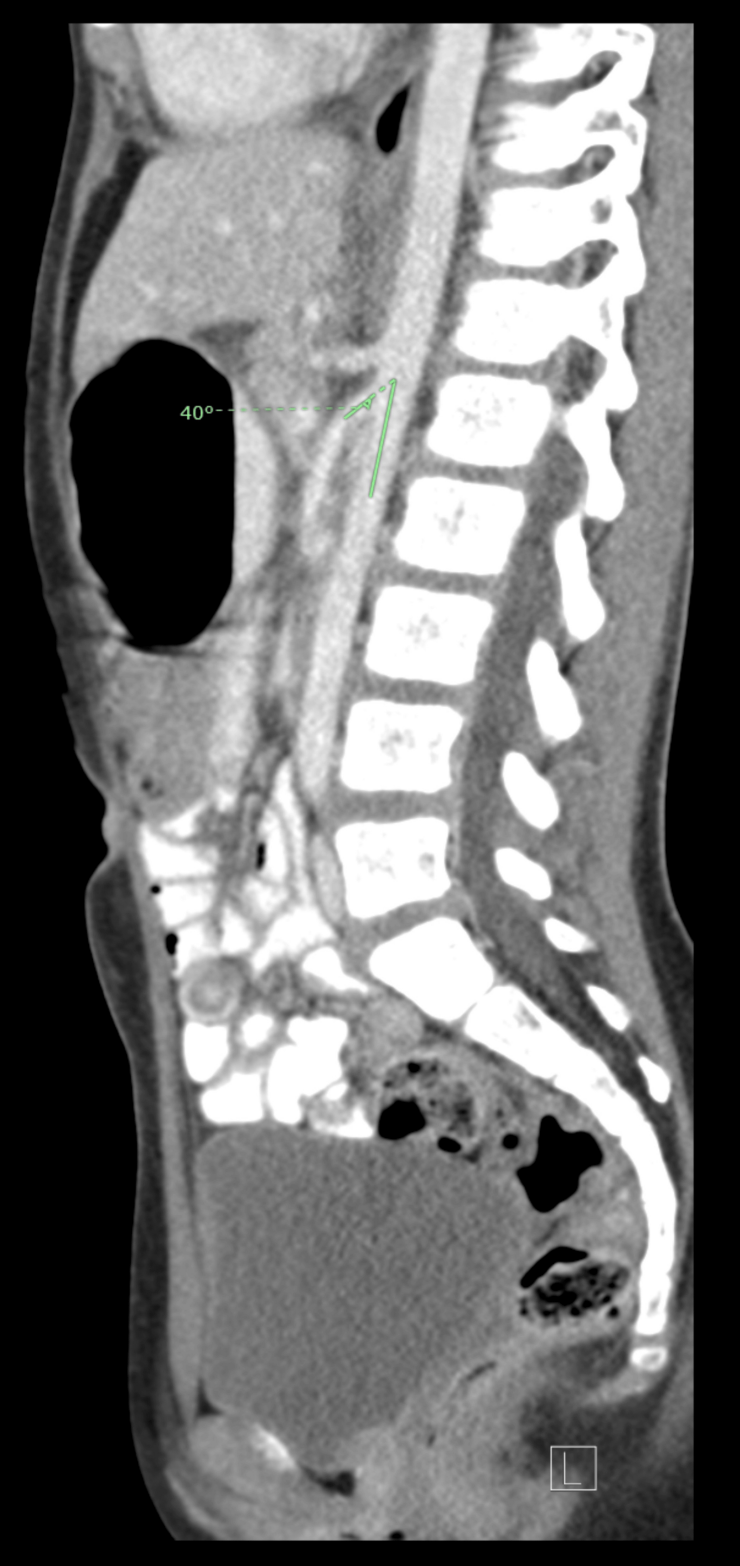
Sagittal CT showing collapsed duodenum between the aorta and SMA with narrowed angle SMA, superior mesenteric artery

An initially seven-year-old girl, who turned eight during her hospital admission, had a past medical history of transient synovitis at age two and a mild COVID-19 infection a year prior. She presented with symptoms of a dry throat, abdominal pain, and vomiting. In the emergency department, her vital signs were normal; however, laboratory results indicated elevated white blood cell count, hemoglobin, hematocrit, neutrophils, blood urea nitrogen, and C-reactive protein (Table 1). Urinalysis revealed proteinuria and ketonuria (Table 2). An abdominal CT scan showed normal findings except for a dilated stomach and proximal duodenum. Specifically, the axial CT image (Figure 1) revealed a dilated proximal duodenum with an air-fluid level, while the sagittal CT image (Figure 2) demonstrated a collapsed third portion of the duodenum between the aorta and the SMA, along with a slightly narrowed aortomesenteric angle. At this stage, management was supportive, including ondansetron and polyethylene glycol laxatives.

**Table 1:** Laboratory results before and after treatment with normal reference ranges

Labs	Before treatment	After treatment	Normal range
C-reactive protein	17.54 mg/l	4.99 mg/l	0-10 mg/l
Blood urea nitrogen	23 mg/dl	15 mg/dl	5-19 mg/dl
Hemoglobin	16.6 g/dl	11.5 g/dl	11.5-14.5 g/dl
Hematocrit	49.20%	37.10%	34-43%
White blood cell count	19.6 × 10^9^/l	12.3 × 10^9^/l	4-12× 10^9^/l
Creatinine	0.69 mg/dl	0.46 mg/dl	0.4-1 mg/dl
Aspartate aminotransferase	50 unit/l	21 unit/l	5-50 unit/l
Alanine aminotransferase	62 unit/l	13 unit/l	5-50 unit/l
Erythrocyte sedimentation rate	7 mm/hr	2 mm/hr	0-12 mm/hr

**Table 2:** Urinalysis results before and after treatment with normal reference ranges

Urinalysis	Before treatment	After treatment
pH	5.5	7
Hemoglobin	Negative	Negative
Protein	1+	Negative
Glucose	Negative	Negative
Ketones	4+	Negative
Bilirubin	1+	Negative
Leukocyte esterase	Negative	Negative
Nitrite	Negative	Negative

**Figure 3:**
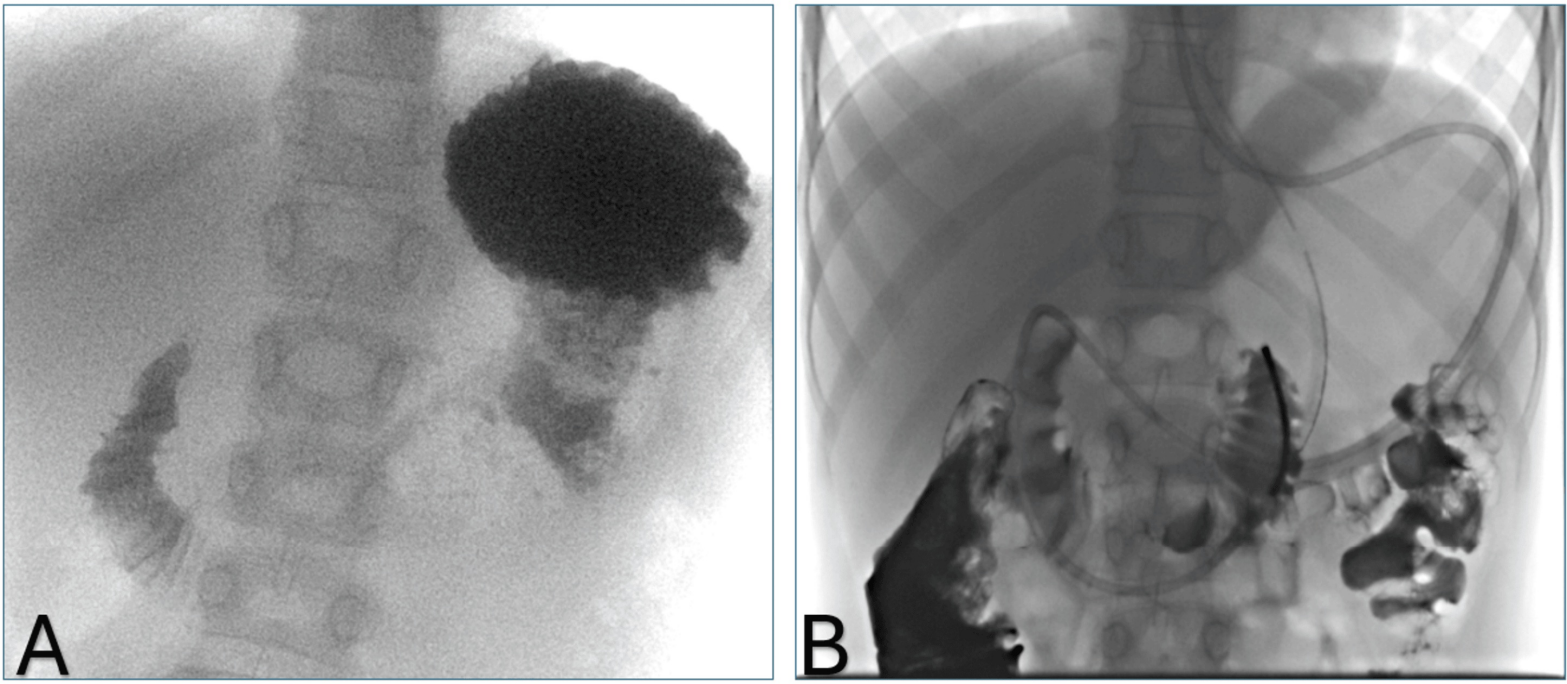
(A) Upper GI fluoroscopy without NJ tube. (B) Upper GI fluoroscopy with NJ tube Upper GI fluoroscopy revealed an obstruction in the third part of the duodenum near the SMA, preventing the passage of enteric contrast (A). Subsequent fluoroscopic placement of an NJ tube demonstrated that while the narrowed area could be crossed, it was achieved with significant difficulty (B). NJ, nasojejunal; SMA, superior mesenteric artery

The patient returned after two days with worsening symptoms, particularly increased vomiting, leading to her admission. Laboratory results remained unchanged. An upper GI) fluoroscopy indicated an obstruction at the level of the third portion of the duodenum near the SMA, with enteric contrast flow impeded and not relieved by repositioning maneuvers (Figure 3). Subsequent fluoroscopic placement of a nasojejunal (NJ) tube demonstrated that the narrowed site could be crossed, suggesting partial patency of the third portion of the duodenum, despite multiple previous failures to insert the NJ tube. The exact cause of the narrowing - whether due to inflammation or a low aortomesenteric angle - remained unclear, and it is possible that both factors contributed.

**Figure 4:**
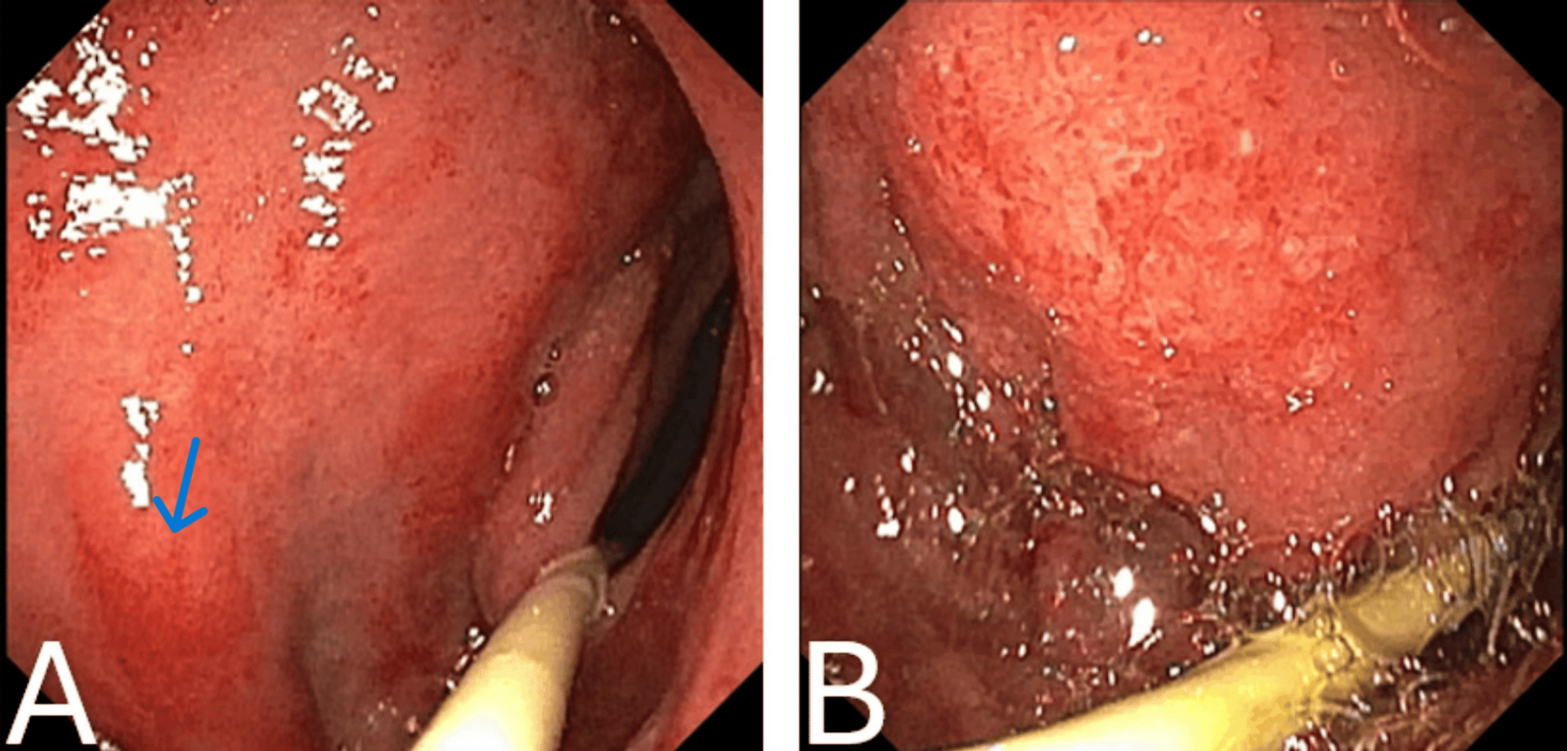
(A) Duodenal bulb. (B) Second portion of the duodenum Endoscopy revealed erythema, mucosal irregularities, and superficial erosions in the duodenal bulb, indicative of duodenitis (A). The second portion of the duodenum displayed diffuse erythema and a granular appearance, suggesting inflammation (B).

**Figure 5:**
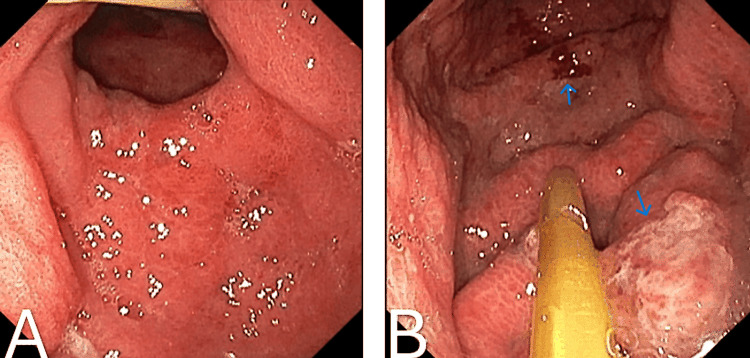
(A) Pre-pyloric stomach. (B) Gastric body Endoscopy revealed significant erythema, mucosal irregularity, and ulceration in the pre-pyloric stomach (A) and the gastric body (B).

**Figure 6:**
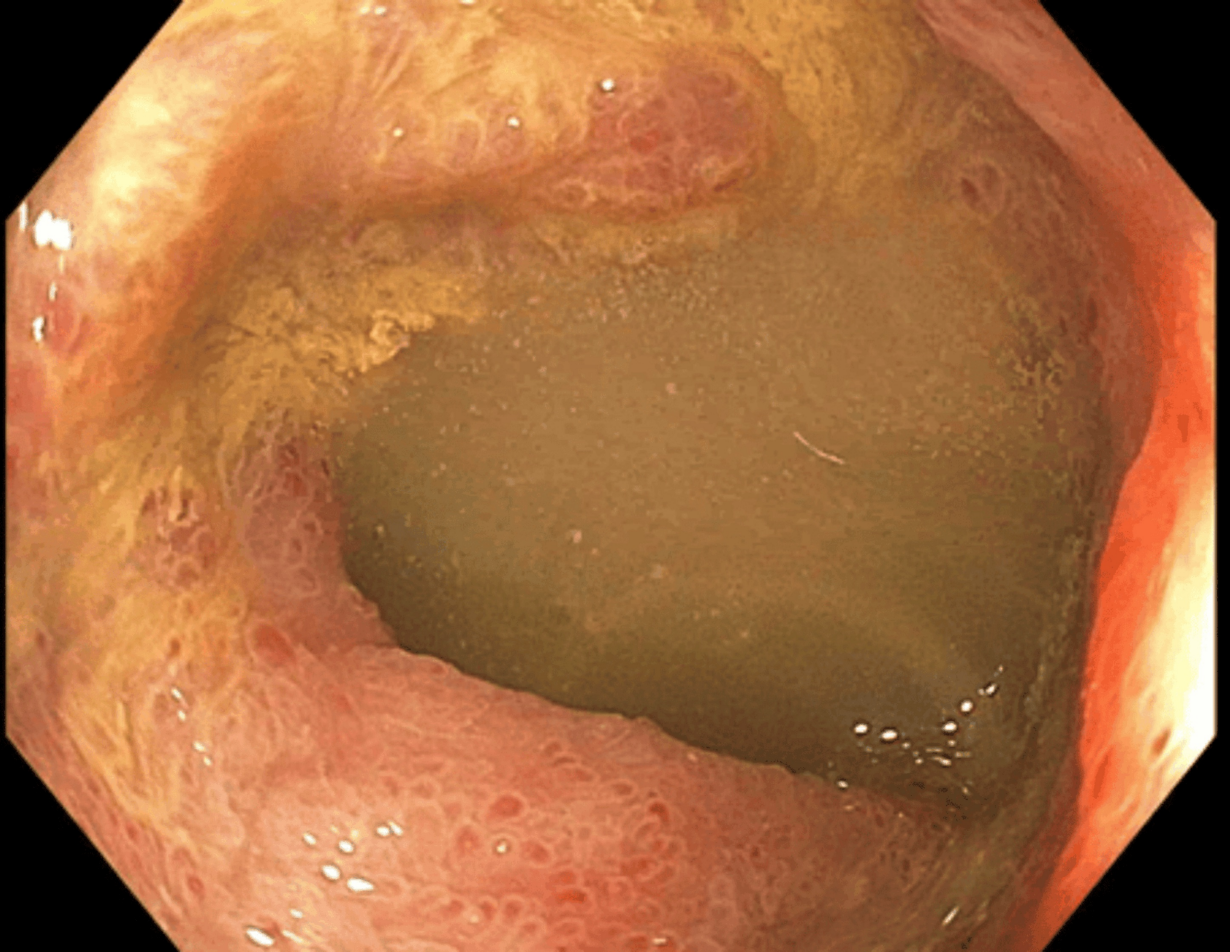
Terminal ileum

Endoscopic examination of the duodenum revealed marked erythema and mucosal irregularity in the duodenal bulb, with areas suggestive of superficial erosion, consistent with active duodenitis. The second portion of the duodenum displayed diffuse erythema and a granular mucosal appearance, indicating significant inflammation (Figure 4). Additionally, endoscopy identified significant erythema, mucosal irregularity, and ulceration in both the gastric body and the pre-pyloric stomach (antrum). The gastric body showed pronounced ulceration, which correlated with histopathological findings of mild chronic gastritis. Similarly, the pre-pyloric stomach exhibited pronounced inflammation and multiple ulcerations, consistent with mild chronic gastritis on histopathology (Figure 5). Examination of the terminal ileum revealed mucosal erythema, indicative of mild inflammation. Histopathology confirmed mild chronic active inflammation with eosinophils and neutrophils present in the lamina propria and surface epithelium (Figure 6). The colon appeared normal throughout. The patient was managed with pantoprazole, nasogastric tube decompression, NJ tube feeding, and intravenous fluids.

**Figure 7:**
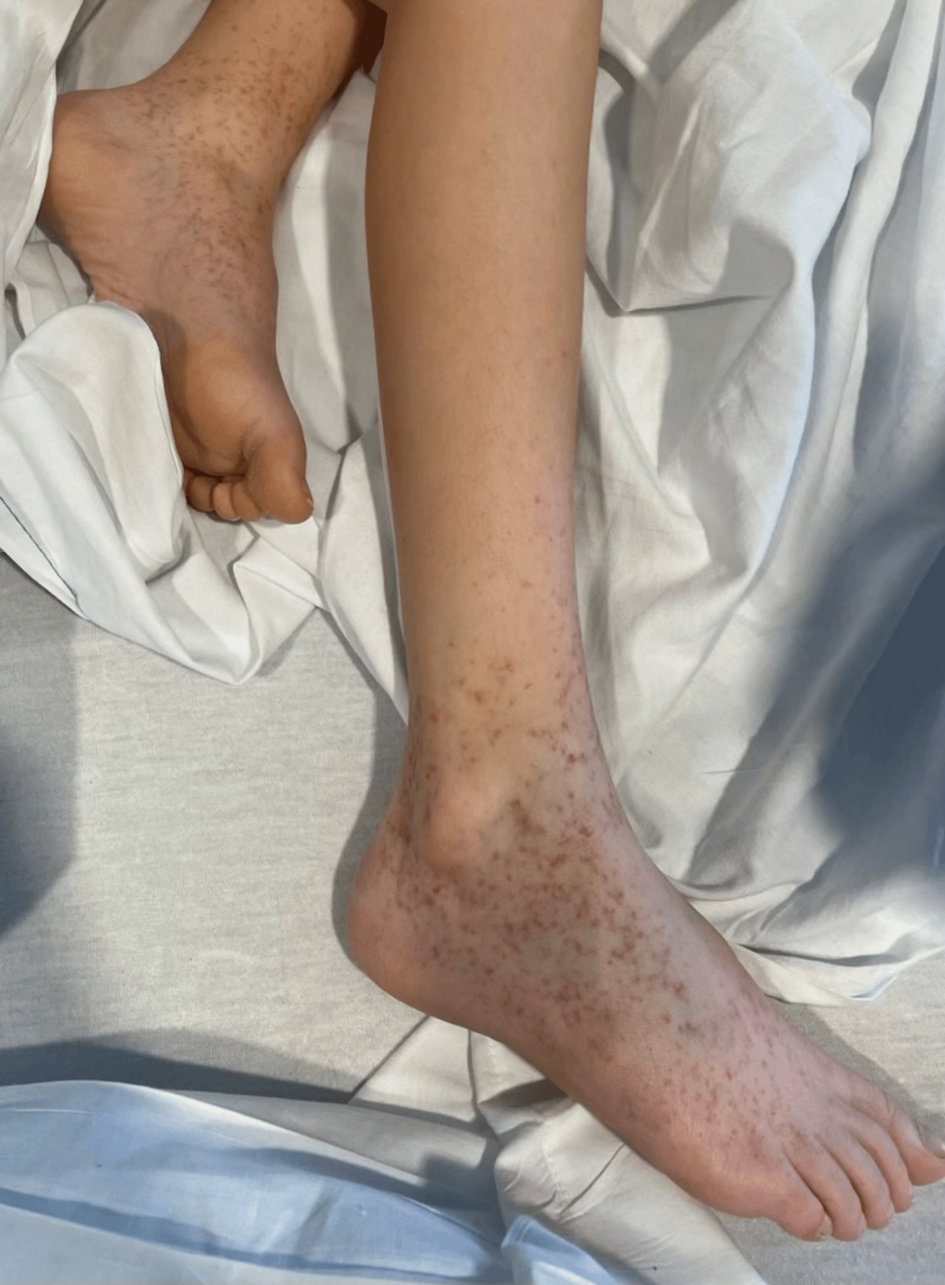
Palpable purpuric rash on the feet and ankles

About one week later, the patient developed rashes on her feet and ankles, consistent with leukocytoclastic vasculitis (LCV) (Figure 7). After consultations with rheumatology and dermatology specialists, a biopsy was deferred due to the typical presentation of the LCV rash. A workup was conducted to rule out other conditions such as granulomatosis with polyangiitis and systemic lupus erythematosus. Laboratory tests were negative for Jo-1 antibodies, dsDNA antibodies, ENA Smith, ENA RNP, ENA SSA, SCL-70, centromere antibody, ENA SSB, antinuclear antibody, antineutrophil cytoplasmic antibody, myeloperoxidase, HLA-B27, and HLA-B51. The antistreptolysin O titer was normal, and the throat culture for streptococcus was negative. Given the presence of the skin rash, GI symptoms, and proteinuria, a diagnosis of HSP with associated SMA syndrome was highly likely.

Initially, we were concerned about using steroids due to the risk of GI perforation and the limited literature supporting their use. However, after reviewing the literature, we found that steroids had been successfully used to treat SMA syndrome associated with HSP in a few cases [5,6]. After discussing the benefits and risks with the family and consulting with specialists in surgery and gastroenterology, we decided to administer intravenous Solu-Medrol (methylprednisolone).

The patient showed significant improvement in her rheumatologic symptoms and SMA syndrome after just three days of intravenous Solu-Medrol, which allowed for the removal of the nasogastric and NJ tubes. Despite initial challenges, including multiple failures of NJ tube insertion, the introduction of total parenteral nutrition was crucial in stabilizing her condition and facilitating weight gain. Combined with steroid therapy, this approach marked a turning point in her recovery, leading to a successful transition to oral feeding and eventual discharge on a tapering regimen of oral steroids.

At 18 months of follow-up, no relapses were noted. The patient remained free of GI symptoms, such as pain or vomiting, and had no recurrence of the rash. Additionally, her C-reactive protein and complete blood count normalized (Table 1). Urinalysis revealed no proteinuria or hematuria, and she continues to maintain normal blood pressure, blood urea nitrogen, and creatinine levels (Table 1, Table 2).

## Discussion

The eight-year-old girl initially presented with symptoms of intestinal obstruction that did not respond to conservative management. Subsequent diagnosis revealed that she had SMA syndrome in the context of HSP. Treatment with intravenous Solu-Medrol (methylprednisolone) and prednisone led to complete resolution of both SMA and HSP symptoms without relapse.

A case report by Harada et al. (2007) described a similar case involving a five-year-old girl diagnosed with HSP who exhibited typical symptoms such as purpura, abdominal pain, and arthralgia. Physical examination showed upper abdominal tenderness without guarding and normal bowel sounds. Abdominal ultrasonography revealed an edematous duodenal wall (6 mm) and a reduced aortomesenteric distance and angle, indicative of SMA syndrome. Prednisolone therapy for 10 days resulted in resolution of symptoms and a reduction in duodenal wall edema to 2.7 mm. Within a month, the edema completely resolved, with a wall thickness of 2.0 mm [5].

Harada et al. (2011) conducted a case-control study comparing 12 HSP patients with 48 matched controls. They found that HSP patients had significantly smaller aortomesenteric angles, reduced aortomesenteric distances, and higher obesity indices. All HSP patients met the ultrasound criteria for SMA syndrome, compared to only 10 controls (100% vs. 20.8%, p < 0.001) [6]. Both studies by Harada et al. suggest that duodenal involvement leading to SMA syndrome can be a manifestation of HSP. They also noted that treatment with prednisone led to full recovery in HSP patients with SMA syndrome. Similarly, our case demonstrates complete recovery with steroid treatment.

This report reinforces the need to consider SMA syndrome as a potential complication of HSP. Addressing the underlying HSP with steroids is crucial for resolving SMA syndrome. Typically, SMA syndrome is managed conservatively with parenteral nutrition, and surgical options like laparoscopic duodenojejunostomy are considered if conservative measures fail [4]. In our case, surgery would likely have been ineffective due to the ongoing HSP.

Our case highlights the importance of recognizing underlying conditions like HSP that may contribute to SMA syndrome. Early intervention with steroid therapy and total parenteral nutrition was pivotal in the patient’s recovery, facilitating weight gain and the ability to tolerate oral foods. Early steroid treatment not only alleviates extrarenal symptoms such as abdominal and joint pain but also influences the course of renal involvement in HSP patients [7]. The resolution of SMA syndrome with steroid treatment suggests that it was a consequence of duodenitis caused by HSP. Notably, bowel symptoms may precede the classic purpuric rash of HSP, as observed in this case.

## Conclusions

This case report highlights the rare occurrence of SMA syndrome as a complication of HSP in an eight-year-old girl. Treatment with intravenous Solu-Medrol resulted in significant improvement of both SMA syndrome and HSP symptoms. Our findings suggest that SMA syndrome may be an aspect of the clinical spectrum of HSP, and prompt corticosteroid therapy can be effective in managing both conditions. We recommend that physicians consider corticosteroid treatment in similar cases, although further research is needed to establish definitive guidelines regarding the benefits, dosing, and duration of such therapy. Additionally, it is crucial to consider underlying HSP when diagnosing SMA syndrome in children.
